# Clinical Therapeutic and Management Strategies for Epilepsy: Psychological Aspects in Children with Epilepsy and Their Parents

**DOI:** 10.3390/jcm15041670

**Published:** 2026-02-23

**Authors:** Hideaki Kanemura

**Affiliations:** Department of Pediatrics, Toho University Medical Center Sakura Hospital, 564-1 Shimoshizu, Sakura 285-8741, Chiba, Japan; hideaki.kanemura@med.toho-u.ac.jp; Tel.: +81-43-462-8811

**Keywords:** stigma, fatigue, headache, seizure activity, educational support, quality of life (QOL)

## Abstract

Epileptic activities can affect various aspects including neuropsychological and social functions, which lead to reductions in quality of life (QOL) for epileptic children. Social function in epileptic children can be negatively impacted due to emotional instability, including stigma associated with having epilepsy. The parents of children with refractory epilepsy could experience severe anxiety regarding clinical seizures in their children, and this severe parental anxiety state could lead to adaptable dysfunction in their children. Moreover, associations between epilepsy and fatigue or headache are well known to clinicians but insufficiently understood. A deeper understanding of these issues in epilepsy could be important for managing the clinical course and treatment regimen. Seizure activities could be associated with impaired neuropsychological/social functions as represented by stigma, fatigue, and headache. Seizure severities are thus important QOL-related factors in terms of neuropsychological and social issues in epileptic children. A relationship between current seizure activity and perception of stigma could be evident in epileptic children. Seizure activity represented as frequent seizures could also be related to fatigue and seizure-associated headache, which can lead to reduced QOL in children with epilepsy. In addition, seizure-related factors in epileptic children including frequent seizures could lead to a greater perception of stigma among their parents. Meanwhile, educational interventions about epilepsy for parents could reduce negative emotional influences when caring for children presenting with seizure attacks. Therapeutic management thus appears desirable to achieve better seizure control and establish educational support for parents, aiming to improve QOL in children with epilepsy.

## 1. Introduction

Seizure activities can affect various aspects, including neuropsychological and social functions, which can reduce quality of life (QOL) in epileptic children. In particular, stigma could be a principal psychological issue for adults and children with epilepsy. In general, stigma and exclusion are considered common features of epilepsy, representing principal issues in the psychosocial problems associated with the condition. Stigma could deteriorate social functions, including relationships around the development and maintenance of self-respect, leading to unfavorable outcomes such as isolation, high rates of unemployment and remaining unmarried, and delayed intervention [1]. Epilepsy is a disorder with social dysfunctions and should therefore not be regarded solely as a neurological disorder.

The majority of epileptic children can achieve favorable outcomes. However, epileptic children generally experience a higher risk of neuropsychological and social dysfunction than those without epilepsy. In particular, epilepsy can stigmatize adolescents and disturb psychosocial development in terms independence, self-esteem, peer relations, mood, and cognition due to their psychological vulnerability [2]. Unfavorable influences on social function may arise in epileptic children due to emotional instability, including the stigma associated with having epilepsy; so, epilepsy can have major impacts on various aspects of a child’s life [3].

Meanwhile, parenting-related stress can lead to parenting dysfunction [4,5]. Epilepsy in children, particularly refractory epilepsy, involves a high risk of parenting dysfunction [6,7,8,9]. Parents of children with refractory epilepsy could have severe anxiety regarding clinical seizures in their children, and this severe parental anxiety could lead to adaptable dysfunction in their children [10]. Accordingly, the clinical manifestations or severity of epilepsy in children may have major impacts on QOL from the perspective of parenting stress [11].

On the other hand, fatigue can also lead to reduced QOL among individuals with chronic neurological diseases and disorders, including epilepsy [12,13,14,15]. Fatigue is regarded as a mental and/or physical state of extreme and persistent weakness, tiredness, or exhaustion [15]. The prevalence of fatigue among individuals with chronic medical conditions is high, but fatigue has been disregarded in the evaluation and management of epileptic children [13]. Although few studies have investigated the problem of fatigue, fatigue could induce seizures [16,17,18], so a better understanding of this issue in epileptic children could be important for the management of the clinical course and treatment regimens.

Both epilepsy and headache are common paroxysmal disorders. A relationship between these two disorders is well known to clinicians, but insufficiently understood. Evaluation and management of headaches associated with seizures is often disregarded at the time of obtaining the medical history, because other symptoms including impairment of consciousness or motor phenomenon are predominant in the information. Headache could be evident in association with seizures as a preictal, ictal, postictal, or inter-ictal phenomenon but tends to be disregarded because the clinical features of seizure are very serious [19]. The pathophysiology of seizure-associated headaches remains unclear due to a lack of consideration. Accordingly, the presence of seizure-associated headaches and risk factors in epileptic children needs to be considered.

The frontal cortex matures over a long period of time and is highly vulnerable due to various factors. Frontal regional damage in children negatively affects the maturation and organization of this area, contributing to neuropsychological impairments including emotional instability [20]. Previous reports have indicated that severe seizures as represented by frequent or prolonged seizures can cause damage to the developing brain [20,21,22,23]. The results of several studies have suggested that seizure severity could lead to impairments of neuropsychological functions as represented by stigma, fatigue, and headache. Moreover, these QOL-related factors could lead to hindrance of therapeutic adherence in epileptic children (Table 1). Seizure severity is thus an important QOL-related factor in terms of social and neuropsychological aspects in children with epilepsy.

This review is conducted as a narrative review based on current accumulated knowledge. This review is based on published works, existing medical texts, and a search of PubMed and MEDLINE for articles with the keywords “quality of life (QOL)”, “stigma”, “fatigue”, “headache”, “epilepsy”, and “children” between January, 1975, and September, 2025. More than 500 articles were originally identified, and a shortlist of core published work of possible relevance to this review was prepared after consideration. Only articles published in English were considered.

## 2. Can Epileptic Activities Affect the Emotional State in Children with Epilepsy?

### 2.1. Relationship Between Seizure Activities and Perception of Stigma

Most epileptic children can obtain favorable outcomes with clinical seizures. However, individuals with epilepsy, including children, could be at significant risk of neuropsychological impairments and tend to show social dysfunctions more frequently than those without epilepsy. Clinical seizures in most children cannot go on indefinitely, but unforeseen events create a sense of fear among the public regarding epilepsy [24]. In particular, epilepsy could stigmatize young people and contribute to neuropsychological and social dysfunction [2]. Social identity in epileptic children could thus be negatively affected due to the stigma associated with having epilepsy. The social impact of stigma could indeed prove more significant than the actual neurological condition itself in some cases [23].

The perception of epilepsy-related stigma seems to be different in low-income countries compared with high-income countries. The perception of stigma is more severe in low-income countries. However, the patients’ share of medical costs remains the same in low-income and high-income populations due to the medical assistance program for children in Japan. Accordingly, differences in the perception of epilepsy-related stigma are unlikely to arise in Japan.

**Figure 1:**
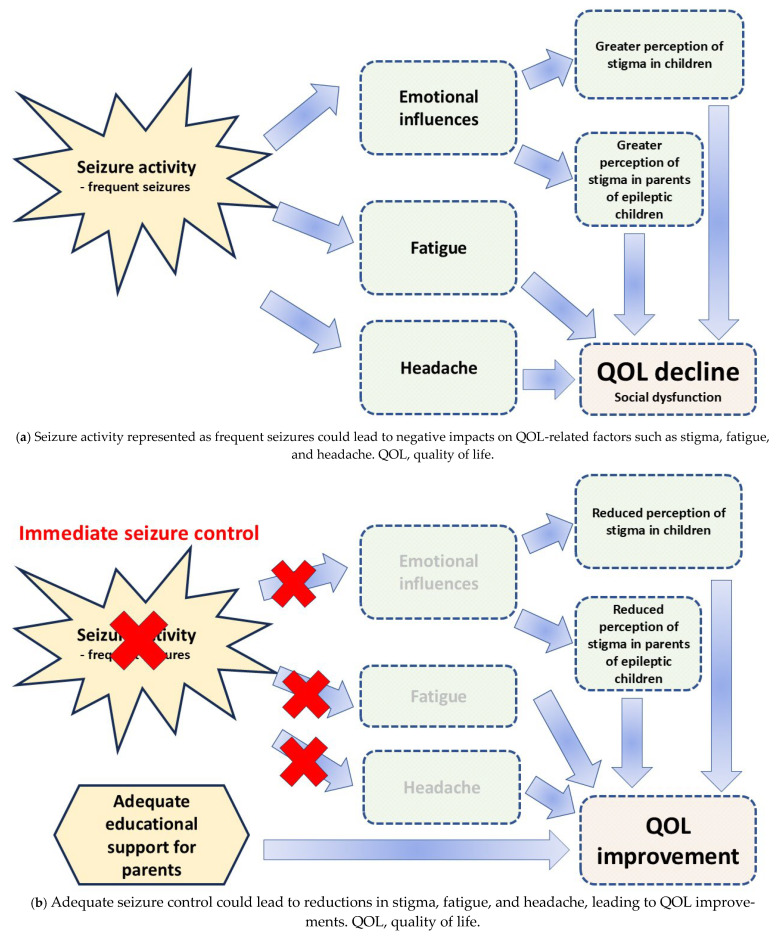
Association between seizure activity and QOL reduction.

The principal aims of epilepsy management should be established so that children with epilepsy can maintain a seizure-free state with well-tolerated treatment [25]. Various studies also suggest that a reduction in negative aspects, including stigma in epileptic children, seems to be an important aim of treating a patient [25,26]. How clinical seizures can influence the perception of stigma in epileptic children remains insufficiently investigated. The perception of stigma can depend on national characteristics and cultures, so this issue needs to be assessed by country or region. In a previous study, the relationship between seizure activities and perception of stigma was evaluated using the Child Stigma Scale (CSS) [27] in Japanese epileptic children between 12 and 18 years old [28]. On the CSS, children rate how often they feel or experience stigma using items scored on a 5-point scale: 1, “never”; 2, “not often”; 3, “sometimes”; 4, “often”; or 5, “very often”. Scores are summed and divided by the number of items scored, as detailed by Austin et al. [27]. On the CSS, higher scores indicate a greater degree of stigma. The results of that study revealed that children presenting with frequent seizures recognized themselves as significantly more stigmatized compared to those in a less frequent or seizure-free state (*p* < 0.01) [28] (Table 2). Such findings suggest a relationship between current seizure activity and perception of stigma (Figure 1a). Children with fewer seizures could have a lower perception of stigma.

### 2.2. Epileptic Activities Including EEG Abnormality and Perceived Stigma: Are They Related?

Associations between neuropsychological functions including emotional state and findings from electroencephalography (EEG) have been indicated. Frontal asymmetrical abnormality on EEG is involved in both the susceptibility to react to emotional stimulation and variations in emotional state [29]. Frontal asymmetrical EEG abnormality can thus lead to neuropsychological impairments, including depression and anxiety [30]. Moreover, neuropsychological dysfunctions including behavioral impairments in neurodevelopmental disorders such as attention-deficit/hyperactivity disorder or autism spectrum disorder could be based on the frequency of frontal interictal epileptiform discharges (IEDs) in some children [31]. Abnormalities on EEG in frontal regions are thus associated with emotional and behavioral functions. The results from a previous study showed that children with epilepsy presented with significantly higher CSS scores than non-epileptic children (*p* < 0.01) [32]. Moreover, a group with frontal IEDs revealed higher CSS scores for all questions compared to scores from other regional IED groups (*p* < 0.01). That study revealed that children presenting with frontal IEDs perceived a stronger degree of stigma than those presenting with non-frontal IEDs (*p* < 0.01) [32]. These observations show a relationship between frontal IEDs and a more significant perception of stigma. Frontal EEG abnormality can function as an emotional mediator including perception of stigma in epileptic children.

### 2.3. Seizure Activities and Fatigue in Epileptic Children: Are They Related?

Among clinical, statistical, and social factors, fatigue is considered to reduce the QOL of epileptic patients [13]. Fatigue can also induce clinical seizures [16]. As fatigue can trigger seizures [17,18], a better comprehension of fatigue among children with epilepsy is very important in supporting effective management of the clinical course and treatment regimens. Fatigue is a frequent complaint in general and highly prevalent in epileptic patients, but little consideration has been given to fatigue in the assessment and management of epileptic children [13]. A previous study evaluated the association between seizure activity and the degree of fatigue in epileptic children using the Fatigue Severity Scale (FSS) [33]. The FSS is constructed using nine items that rate fatigue on a scale from 1 (strongly disagree) to 7 (strongly agree) [34]. Epileptic children are required to use a 7-point scale (1 to 7) to rate how often over the previous week they felt the emotions shown by the nine items. After summation of the scores, the total score is divided by 9, resulting in values from 1 to 7 [34]. A higher score reflects a greater level of fatigue. That study showed that mean FSS scores were significantly higher in children with epilepsy than in those without (4.40 vs. 1.55, respectively; *p* < 0.0001). In addition, multiple linear regression analysis revealed seizure frequency as the only clinical manifestation significantly associated with fatigue (*p* < 0.0001) [33]. The same study showed that children with uncontrolled seizures experienced greater fatigue levels than those with well-controlled seizures (*p* < 0.0001) [33]. These findings indicate that frequent seizures could lead to greater levels of fatigue (Table 2). Seizure activity represented by frequent seizures could correlate with fatigue level in children with epilepsy. Fatigue levels should thus be taken into consideration in children with epilepsy (Figure 1a).

### 2.4. Seizure Activities and Headache in Epileptic Children: Are They Related?

Both epilepsy and headache are common underlying mechanisms in functional and paroxysmal disorders. Headaches, including migraines and headaches associated with epilepsy, are chronic disorders that can have a variety of underlying causes and symptoms, and patients with epilepsy often experience epilepsy-related headaches, including both seizure-related and seizure-unrelated headaches. However, the underlying mechanism of peri-ictal headaches in epilepsy is insufficiently clarified. In general, headaches in children are not yet fully diagnosed and are not appropriately regarded as a medical issue by clinicians [35]. In a previous study of 98 epileptic children and adolescents between 5 and 18 years old, 34 (34.7%) complained of epilepsy-related headaches [36]. Moreover, seizure frequency was 4.1 times per year in children with headaches and 1.3 times per year in those without headaches. That study concluded that epilepsy-related headaches were more frequent in epileptic children than in children with uncontrolled seizures [36] (Table 2). These findings indicate that seizure activity represented by frequent seizures could be related to the presence of epilepsy-related headache, which can lead to reduced QOL among children with epilepsy (Figure 1a).

## 3. Can Seizure Activities Affect the Emotional State of Parents of Epileptic Children?

### 3.1. Fears and Anxieties in Parents of Children Presenting with First Febrile Seizures

Febrile seizures (FSs) represent a highly prevalent seizure disorder in childhood and show good outcomes [37]. However, parents of children with FSs may interpret the symptoms as a very shocking occurrence [38]. Insufficient knowledge concerning FSs among parents could lead to misunderstandings about the possibilities of undesirable outcomes, including life-threatening illness, brain damage, later development of epilepsy, and tongue biting [39,40,41]. In fact, a child presenting with convulsive symptoms can shock inexperienced parents who may think that the child is at a high risk of death [42,43]. Although FS is recognized as a highly prevalent disorder with good prognosis [44], significant differences exist between the harmful emotional state of parents and the comparatively insignificant clinical approach of clinicians. Parental fear and anxiety regarding FS can be a critical issue, with serious negative effects on daily familial life [42]. Pediatricians and general practitioners may not even be aware of the stress faced by parents of children with FS [44]. In a previous study, 70% (55/78) of parents of children with simple FS mentioned that they had considered that the child would die during a convulsive attack or may be severely injured [45]. In another study, parental stress and stigma were evident within 3 months after convulsive episodes in their children [46]. Such studies indicate that parents experience inappropriate thoughts and actions due to fears and anxieties, as perceived by many parents in cases where their child is experiencing convulsions.

### 3.2. Seizure Activity and Perception of Stigma Among Parents of Epileptic Children: Are They Related?

It is important that pediatric epileptologists recognize the influence of stigma accompanying epilepsy given the necessity for developing and implementing management strategies to achieve better psychosocial health for both epileptic children and their parents. Stigma is a general aspect associated with epilepsy, with substantial negative influences on epileptic children, their parents, and their QOL. Epileptic patients tend to consider the perception of stigma as one of their biggest problems [47]. Epileptic activity and severity also represent major factors contributing to parenting-related stress [11]. In a previous study [48], the authors assessed parental stigma in association with epilepsy among parents of Japanese children with epilepsy using the Parental Stigma Scale (PSS). On the PSS, parents are required to rate five items in the questionnaire using a 5-point scale: 1, strongly disagree; 2, disagree; 3, neither; 4, agree; and 5, strongly agree. The ultimate score is calculated as the mean of each score, as described by Austin et al. [26]. In that study, PSS scores were higher in the parents of epileptic children than in the parents of non-epileptic children [48]. Moreover, that study also showed that seizure activity represented by frequent seizures (defined as seizures at least once a month) correlated with greater perceptions of stigma in multiple regression analysis (*p* = 0.0036). These findings suggest that seizure activity as represented by frequent seizures in children leads to greater perceptions of parental stigma [48]. However, other studies have indicated that parenting-related stress did not correlate with seizure frequency in a direct manner [6,9,13]. Papazoglou et al. showed that the presence of seizures, regardless of frequency, could lead to adaptive dysfunction in epileptic children [49]. In contrast, a previous study revealed that frequent seizures could affect the perception of stigma among epileptic children [28]. In addition, the perception of stigma could lead to adaptive dysfunction in children [27]. These findings indicate that frequent seizures are associated with greater parental stress (Table 2). Seizure-related factors in epileptic children as represented by frequent seizures thus lead to a greater perception of stigma among parents (Figure 1a).

## 4. Management and Therapeutic Approaches for Children with Epilepsy

### 4.1. Is Immediate Suppression of Clinical Seizures Needed? Thinking from Cognitive and Behavioral Perspectives

As described above, seizure activity could be associated with several QOL-related factors, including stigma, fatigue, and headache. In addition, seizure activities represented by frequent and prolonged seizures could lead to neuropsychological impairments, including cognitive dysfunction and behavioral disturbances [20,31]. Moreover, recovery of prefrontal lobe growth relies on periods of high seizure activity. Previous studies showed that growth of the prefrontal lobe could recover immediately in children with a shorter period of high seizure activity [20]. In contrast, this growth disturbance could be prolonged in children with a longer period of high seizure activity [20]. Further, prolonged seizures in children could lead to cognitive declines and behavioral disturbances due to impaired growth of the prefrontal lobe [20]. Accordingly, therapeutic management is warranted to reduce clinical seizures immediately and thus avoid neuropsychological impairments in epileptic children.

### 4.2. Is Immediate Suppression of Clinical Seizures Needed? Considerations of Emotional State in Epileptic Children

Regarding this issue, immediate suppression of clinical seizures could be beneficial to reduce emotional influences, including stigma. In a preliminary study, the relationship between reductions in clinical seizures and CSS scores was evaluated in a patient with frontal lobe epilepsy (FLE).

The clinical course of this patient is outlined briefly as follows. The patient was a 14-year-old boy in whom psychomotor development was not significant. At 10 years old, he had developed epileptic seizures such as rightward deviation of the head and eyes. Seizures were brief (10–25 s), frequent, and stereotypic. Family history of epilepsy was absent. MRI of the brain revealed no significant findings. Left frontopolar IEDs superimposed on normal background activity were evident on EEG. FLE was diagnosed in accordance with seizure type and the findings on EEG, including IED location. Carbamazepine was initiated, but failed to control seizures. Zonisamide was added to the treatment regimen, but control of seizures was not achieved. Carbamazepine was thus replaced with levetiracetam (LEV), leading to partial improvement in seizures. Treatment was then changed to perampanel (PER) combined with LEV and control of seizures was achieved.

Serial CSS scores were obtained from this patient every 3 months after seizure onset. The CSS score was 1.9 at seizure onset. CSS scores then increased to a maximum of 4.4 at 6 months after seizure onset, in parallel with frequent seizures. However, CSS scores decreased gradually with achievement of increasing seizure control. The CSS score at 24 months after onset had decreased to 1.5. These results indicate that changes in CSS scores were associated with seizure frequency (Table 2). Therapeutic management is thus desirable to immediately reduce clinical seizures and prevent negative emotional influences as represented by the perception of stigma in epileptic children (Figure 1b).

### 4.3. Is Immediate Suppression of Clinical Seizures Needed? Considerations of Emotional State in Parents of Epileptic Children

Immediate suppression of clinical seizures could also be beneficial for reducing negative emotional influences, including stigma, among parents of children with epilepsy. A preliminary study evaluated the relationship between reductions in clinical seizures and PSS scores in the mother of a child with epilepsy.

The clinical course of this patient is outlined as follows. The patient was a 12-year-old boy in whom psychomotor development was not significant. At 10 years old, he experienced epileptic seizures such as impaired awareness and motion arrest. The duration of each seizure was about 45–90 s. Seizures were stereotypical and repeated frequently. Family history of epilepsy was absent. MRI of the brain revealed no significant findings. Diffuse spike–wave complexes superimposed on normal background EEG activity were evident. LEV was initiated, but seizures showed drug resistance. Focal to bilateral tonic–clonic seizures subsequently appeared. No response to administration of lacosamide was seen. Treatment was changed to PER combined with LEV, leading to immediate improvements in seizures.

Serial PSS scores were obtained every 3 months after seizure onset from the mother of this patient. The PSS score at seizure onset was 2.0. PSS scores increased serially to a maximum of 4.8 at 6 months after seizure onset, in parallel with frequent seizures. However, PSS scores gradually decreased with successful seizure control in her child. The PSS score decreased to 1.6 by 18 months after seizures had stopped. These results indicate that changes in PSS scores could be related to seizure activity as represented by frequent seizures in children (Table 2). Therapeutic management is thus desirable to immediately reduce clinical seizures and prevent negative emotional influences in parents of children with epilepsy (Figure 1b).

### 4.4. Is an Effective Educational Intervention Program Needed for Parents and the Public?

Medical knowledge regarding epilepsy has recently become more widespread. Most epileptic syndromes in children represent benign conditions. However, insufficient comprehension about epilepsy among parents may be exacerbated by misinformation about the risks of brain damage and poor developmental/social outcomes. The cause, associated factors, history of clinical course, and management of epilepsy have been broadly investigated, but parental thoughts and actions after witnessing a seizure attack in their child are not widely known. Enhancing the understanding of parental knowledge and thoughts regarding seizure disorders including epilepsy could improve parent–clinician relationships and enhance the efficacy of the information provided [44]. A previous study examined their thoughts and actions of parents of 78 children who presented with their first simple-type FS [45]. Approximately 77% of parents did not have preceding knowledge about FS. Almost all parents (91%) took the child directly to hospital in an ambulance after a 119 emergency call. In addition, 41% of parents had reported thinking that the child was close to dying due to the seizure attack, and 29% thought that the child was in a serious condition and had suffered severe brain damage [45]. That study also showed that 80% of parents without preceding knowledge of FS were more likely to think that their child was almost dying or in a serious condition, compared with 39% of parents with preceding knowledge. Parents without preceding knowledge about FS more frequently considered the FS as injurious compared to those with preceding knowledge (*p* < 0.03) (Table 2). Parents with preceding knowledge about FS better understood that their child was experiencing FS compared to those without preceding knowledge (*p* < 0.001). Meanwhile, the emotional reaction of the first parent faced with a convulsing child was frequently one of fear and panic. Parents with preceding knowledge about FS managed seizure attacks more adequately than those without this knowledge (*p* < 0.03) (Table 2). These findings suggest that information concerning FS should be provided to all parents [45].

Similarly to FS, knowledge about how to best care for their child with epilepsy could improve the management of epilepsy for parents. Overall expertise concerned with epilepsy among parents and the public is insufficient, and the emotional response of parents to seizure attacks is often serious. However, when parents have appropriate knowledge prior to their child’s first FS, a significant proportion of parents can recall this knowledge and recognize FS, easing their anxiety and encouraging more appropriate actions [45]. Educational interventions about epilepsy for parents could thus reduce negative emotional influences when caring for their children presenting with seizure attacks. Such findings accentuate the significance of acknowledging and addressing parental emotional responses. Reducing emotional influences including stigma and anxiety in parents through adequate education and awareness, changing their perceptions and deepening their understanding, represents a feasible approach to enhancing parenting function. Coordinating an assistant team and an adequate educational intervention for parents could also prove important in the care and management of children with epilepsy (Figure 1b).

Moreover, recently, a smartphone app (Epilepsy Diagnosis Aid) has been developed and validated to be used by Non-Physician Health Workers (NPHWs) in order to confirm the diagnosis of epilepsy. Giuliano et al. showed that the app could represent a valuable instrument that can easily be employed by trained NPHWs to diagnose epilepsy in primary healthcare settings in low- and middle-income countries [50]. Early diagnosis and, consequently, appropriate management with the introduction of new technologies including these devices can improve disease insight among both children with epilepsy and their families.

## 5. Future Perspectives

As shown in various studies, seizure aggravation including frequent and prolonged seizures could lead to reduced QOL in epileptic children. Adequate seizure control may improve QOL for epileptic children from several neuropsychological and social perspectives. However, interpretation of this aspect may need more consideration. Prompt seizure remission could achieve cognitive and behavioral improvements. Meanwhile, improvements or reductions in other issues, including stigma, fatigue, and headache, using antiseizure medication has not yet been sufficiently confirmed. Regarding stigma, increases and reductions in stigma scores of epileptic children and their parents could be associated with seizure activity as represented by seizure frequency. However, this is primarily based on findings from a single case. Accordingly, definitive conclusions cannot yet be drawn. Moreover, associations between reductions in fatigue/headache levels and seizure reduction have not been fully evaluated. Further investigations of a larger cohort are needed to confirm associations between adequate seizure control and improvements in QOL-related factors in children with epilepsy.

In addition, we must consider other factors in QOL in patients with epilepsy such as seizure worry, emotional well-being, medication side effects, social functioning, comorbidity (depression, etc.), social support, and many more in accordance with the QOLIE-31 (QOL in epilepsy). In contrast, this review focuses on seizure control, stigma, fatigue, and headache. The objective of this review was to assess the association between seizure activities and QOL-related factors, focusing on the perception of stigma, fatigue, and headache from current research findings. Thus, this review is based on published works. Based on these previous observations, the therapeutic strategies in children with epilepsy have been discussed in this review. However, we should not ignore the numerous other important factors. Further research will be needed to evaluate the association between seizure activity and other important QOL-related factors.

## 6. Conclusions

Neuropsychological impairments and social dysfunction, including emotional instabilities and behavioral disturbances, are not necessarily present in all children with epilepsy. However, seizure aggravation, including frequent and prolonged seizures, could trigger these negative impacts. Clinicians should concentrate on achieving early seizure remission to prevent such impairments. In addition, coordinating an assistant team and an adequate educational intervention for parents could also lead to reductions in anxiety and stigma among the parents of children with epilepsy. According to the findings obtained from various studies, therapeutic management is desirable to immediately reduce clinical seizures and educational support should be established for parents to improve QOL in children with epilepsy.

## Figures and Tables

**Table 1:** Factors that promote or hinder therapeutic adherence in the pediatric population.

QOL-Related Factors in Association with Social Aspects	Influences of Factors	
	Promote	Hinder
Stigma in children		probable
Stigma in parents	possible (depending on the case)	possible (depending on the case)
Fatigue		probable
Epilepsy-associated headache	possible	
Parental anxiety and fear	possible (depending on the case)	possible (depending on the case)

QOL, quality of life.

**Table 2:** Seizure activity and QOL-related factors in association with social aspects.

QOL-Related Factors in Association with Social Aspects	Findings
Stigma in children	# A relationship could be evident between current seizure activity represented by frequent seizures and perception of stigma. # Children with less seizure could have less perception of stigma. # Increases and reductions in CSS scores could be associated with seizure frequency.
Stigma in parents	# PSS scores in parents of epileptic children were significantly higher than those of non-epileptic children. # Seizure activity represented by frequent seizures (at least once a month) was correlated with greater perceptions of stigma. # Seizure activity represented by frequent seizures in their children could lead to greater perceptions of parental stigma. # Increases and reductions in PSS scores could be related to seizure activity represented by frequent seizures of their children.
Fatigue	# Fatigue Severity Scale scores of the epileptic children were significantly higher than those of the non-epileptic children. # Frequency of seizures was the only significance of clinical manifestations in association with fatigue. # Children in the uncontrolled seizure group presented greater fatigue levels than those in the well-controlled seizure group. # Seizure activity represented by frequent seizures could correlate with fatigue level in epileptic children.
Epilepsy-assocaited headache	# About 35% epileptic children complained of having epilepsy-related headaches. # Epilepsy-related headaches in epileptic children were more frequent in children with uncontrolled seizures. # Seizure activity represented by frequent seizures could relate to the presence with epilepsy-related headaches.
Parental anxiety and fear	# Eighty percent of parents without preceding knowledge concerned with FS were more likely to recognize that the child was almost dying or in serious condition in comparison with 39% of those with preceding knowledge. # Parents without preceding knowledge concerned with FS frequently considered that FS was injurious in comparison with those with preceding knowledge. # Parents with preceding knowledge concerned with FS understood that their child was having an FS at a higher rate than those without preceding knowledge. # Parents with preceding knowledge concerned with FS managed seizure attacks more adequately than those without preceding knowledge.

QOL, quality of life; CSS, Child Stigma Scale; PSS, Parental Stigma Scale; FS, febrile seizure.

## Data Availability

No new data was created.
